# Development and validation of a prognostic nomogram for predicting in-hospital mortality of COVID-19: a multicenter retrospective cohort study of 4086 cases in China

**DOI:** 10.18632/aging.202605

**Published:** 2021-02-09

**Authors:** Li Li, Xiaoyu Fang, Lixia Cheng, Penghao Wang, Shen Li, Hao Yu, Yao Zhang, Nan Jiang, Tingting Zeng, Chao Hou, Jing Zhou, Shiru Li, Yingzi Pan, Yitong Li, Lili Nie, Yang Li, Qidi Sun, Hong Jia, Mengxia Li, Guoqiang Cao, Xiangyu Ma

**Affiliations:** 1 Department of Respiratory Medicine, Daping Hospital, Army Medical University, Former Third Military Medical University, Chongqing, China; 2 Wuhan Huoshenshan Hospital, Wuhan, China; 3 College of Public Health, Southwest Medical University, Luzhou, Sichuan, China; 4 Department of Epidemiology, College of Preventive Medicine, Army Medical University, Former Third Military Medical University, Chongqing, China; 5 Daping Hospital, Army Medical University, Former Third Military Medical University, Chongqing, China; 6 Wuhan Taikang Tongji Hospital, Wuhan, China; 7 The Second Clinical College, Chongqing Medical University, Chongqing, China; 8 Department of Endocrinology, General Hospital of Northern Theater Command, Shenyang, Liaoning, China; 9 NCO School of Army Medical University, Former Third Military Medical University, Shijiazhuang, Hebei, China; 10 Department of Oncology, Daping Hospital, Army Medical University, Former Third Military Medical University, Chongqing, China

**Keywords:** COVID-19, SARS-Cov-2, coronavirus, nomogram, survival

## Abstract

To establish an effective nomogram for predicting in-hospital mortality of COVID-19, a retrospective cohort study was conducted in two hospitals in Wuhan, China, with a total of 4,086 hospitalized COVID-19 cases. All patients have reached therapeutic endpoint (death or discharge). First, a total of 3,022 COVID-19 cases in Wuhan Huoshenshan hospital were divided chronologically into two sets, one (1,780 cases, including 47 died) for nomogram modeling and the other (1,242 cases, including 22 died) for internal validation. We then enrolled 1,064 COVID-19 cases (29 died) in Wuhan Taikang-Tongji hospital for external validation. Independent factors included age (HR for per year increment: 1.05), severity at admission (HR for per rank increment: 2.91), dyspnea (HR: 2.18), cardiovascular disease (HR: 3.25), and levels of lactate dehydrogenase (HR: 4.53), total bilirubin (HR: 2.56), blood glucose (HR: 2.56), and urea (HR: 2.14), which were finally selected into the nomogram. The C-index for the internal resampling (0.97, 95% CI: 0.95-0.98), the internal validation (0.96, 95% CI: 0.94-0.98), and the external validation (0.92, 95% CI: 0.86-0.98) demonstrated the fair discrimination ability. The calibration plots showed optimal agreement between nomogram prediction and actual observation. We established and validated a novel prognostic nomogram that could predict in-hospital mortality of COVID-19 patients.

## INTRODUCTION

Since being publicly characterized as a pandemic by the World Health Organization on March 11^th^, 2020, the coronavirus disease 2019 (COVID-19) has become an urgent threat to global public health [1]. The outbreak of COVID-19 led to a significant increase in demand for hospital beds and medical equipment, and several countries have been confronted with a critical care crisis [2]. Therefore, it is urgently needed to set up clinical prediction models for COVID-19 mortality to stratify the most vulnerable patients, to provide them with the best possible care while mitigating the burden on the whole healthcare system.

A number of studies have identified risk factors associated with poor outcomes in COVID-19 univariate/multivariate analyses [3]. For example, older age, comorbidities, higher sequential organ failure assessment (SOFA) score, lower lymphocyte count and increased d-dimer have been reported to be associated with an increased risk of death for COVID-19 patients [4–6]. Besides, several models have been developed to assist in the prognosis of COVID-19 mortality, including nomogram [7], decision tree [8], score system [9], online tools [10], and computed tomography based scoring rule [11], most of which are still in preprint. However, as pointed out by a recent systematic review [12], despite 23 prognostic models to predict mortality risk in patients with COVID-19 having been reported, none was recommended for use in practice due to several limitations. First, some studies suffered from severe sampling bias which was caused by excluding participants who didn’t reach an endpoint (recovered or died). Second, limited sample size, varied length of follow-up, highly subjective predictors, and lack of external validation. Third, the calibration of the models was rarely assessed. Fourth, the guidelines of transparent reporting of a multivariable prediction model for individual prognosis or diagnosis (TRIPOD) were not complied with, and prediction model risk of bias assessment tool (PROBAST) showed these studies were at high risk of bias.

In the current study, we aimed to establish an effective prognostic nomogram for predicting in-hospital mortality of COVID-19 patients. We presented the details of all 4,086 patients with laboratory-confirmed COVID-19 admitted to the two designated hospitals in Wuhan, Huoshenshan Hospital and Taikang Tongji Hospital, as of April 10^th^. The prognostic nomogram was validated by internal 1,000 bootstrap resampling, internal and external validation cohorts. The performance of the nomogram was measured by Harrel concordance index (C-index) for discrimination and the calibration plot for calibration.

## RESULTS

### Characteristics of the COVID-19 patients

Table 1 presents the demographic and clinical characteristics of the included COVID-19 patients in the development cohort and the validation cohort. Of the 4,086 COVID-19 cases, 98 (2.4%) died. Of the 3,988 discharged patients, the median duration of hospitalization was 14 days (IQR: 9-20). For the 98 died, the median duration from admission to death was 9 days (IQR: 5-17). Males accounted for 50.0% (n=2,043) of the total cases, and the median age was 61 (IQR: 50 to 69). For the severity at admission, 48 (1.2%) were classified as mild, 2,882 (70.5%) as ordinary, 1076 (26.3%) as severe, and 80 (2.0%) as critical. The top five symptoms were fever (70.6%), cough (69.2%), fatigue (5=54.7%), anorexia (52.5%), and short breath (41.3%), respectively. The top five coexisting disorders were hypertension (31.7%), diabetes (14.5%), cardiovascular disease (10.5%), coronary heart disease (7.3%), and Chronic liver disease (5.3%), respectively.

**Table 1:** Demographic and clinical characteristics of the included COVID-19 patients.

**Variables**	**Total (N=4086)**	**Development cohort (N=1780)**	**Internal validation (N=1242)**	**External validation (N=1064)**
Death	98 (2.4%)	47 (2.6%)	22 (1.8%)	29 (2.7%)
Gender, male	2043 (50.0%)	914 (51.3%)	627 (50.5%)	502 (47.2%)
Age (Years old), median (IQR)	61 (50-69)	60 (49-68)	60 (49-69)	62 (51-71)
0-14	3 (0.1%)	2 (0.1%)	-	1 (0.1%)
15-49	1003 (24.5%)	444 (24.9%)	317 (25.5%)	242 (22.7%)
50-64	1505 (36.8%)	690 (38.8%)	465 (37.4%)	350 (32.9%)
≥65	1575 (38.5%)	644 (36.2%)	460 (37.0%)	471 (44.3%)
Severity at admission				
Mild	48 (1.2%)	16 (0.9%)	15 (1.2%)	17 (1.6%)
Ordinary	2882 (70.5%)	1260 (70.8%)	903 (72.7%)	719 (67.6%)
Severe	1076 (26.3%)	478 (26.9%)	312 (25.1%)	286 (26.9%)
Critical	80 (2.0%)	26 (1.5%)	12 (1.0%)	42 (3.9%)
Respiratory rate (times)	20 (18-21)	20 (19-21)	20 (19-21)	16 (11-23)
**Symptoms — no. (%)**				
Fever	2885 (70.6%)	1389 (78.0%)	833 (67.2%)	663 (62.3%)
Cough	2827 (69.2%)	1346 (76.2%)	841 (67.9%)	640 (60.2%)
Fatigue	2237 (54.7%)	1128 (63.9%)	633 (51.1%)	476 (44.7%)
Anorexia	2145 (52.5%)	1047 (59.3%)	644 (51.9%)	454 (42.7%)
Short breath	1686 (41.3%)	931 (52.7%)	527 (42.5%)	220 (20.7%)
Myalgia	1093 (36.7%)	675 (38.3%)	329 (26.6%)	89 (8.4%)
Chest tight	1179 (28.9%)	589 (33.3%)	292 (23.6%)	298 (28.0%)
Expectoration	728 (17.8%)	313 (17.7%)	214 (17.2%)	201 (18.9%)
Dyspnea	327 (8.0%)	112 (6.3%)	81 (6.5%)	134 (12.6%)
Diarrhea	268 (6.6%)	121 (6.8%)	65 (5.2%)	82 (7.7%)
Sore throat	227 (5.6%)	80 (4.5%)	48 (3.9%)	99 (9.3%)
Nausea	121 (3.0%)	58 (3.3%)	30 (2.4%)	33 (3.1%)
Dizziness	107 (2.6%)	47 (2.7%)	29 (2.3%)	31 (2.9%)
Headache	104 (2.5%)	53 (3.0%)	17 (1.4%)	34 (3.2%)
Vomiting	104 (2.5%)	51 (2.9%)	21 (1.7%)	32 (3.0%)
Chill	94 (2.3%)	38 (2.2%)	21 (1.7%)	35 (3.3%)
Hemoptysis	26 (0.6%)	11 (0.6%)	10 (0.8%)	5 (0.5%)
**Coexisting disorders — no. (%)**				
Hypertension	1294 (31.7%)	535 (30.1%)	385 (31.0%)	374 (35.2%)
Diabetes	592 (14.5%)	260 (14.6%)	168 (13.5%)	164 (15.4%)
Cardiovascular disease	427 (10.5%)	152 (8.5%)	129 (10.4%)	146 (13.7%)
Coronary heart disease	300 (7.3%)	114 (6.4%)	96 (7.7%)	90 (8.5%)
Chronic liver disease	215 (5.3%)	109 (6.1%)	84 (6.8%)	22 (2.1%)
Cerebrovascular disease	195 (4.8%)	53 (3.0%)	63 (5.1%)	79 (7.4%)
Respiratory disease	175 (4.3%)	36 (2.0%)	53 (4.3%)	86 (8.1%)
Chronic kidney disease	123 (3.0%)	43 (2.4%)	52 (4.2%)	28 (2.6%)
Tumor	92 (2.3%)	35 (2.0%)	42 (3.4%)	15 (1.4%)
Bronchitis	81 (2.0%)	19 (1.1%)	33 (2.7%)	29 (2.7%)
COPD	60 (1.5%)	15 (0.8%)	15 (1.2%)	30 (2.8%)
**Laboratory findings**				
White-cell count (×10^9^/L)	5.8 (4.7-7.1)	5.7 (4.6-7.1)	5.7 (4.7-7.0)	5.8 (4.8-7.0)
Neutrophil count (×10^9^/L)	3.52 (2.68-4.73)	3.52 (2.67-4.80)	3.46 (2.72-4.48)	3.51 (2.66-4.89)
Lymphocyte count (×10^9^/L)	1.52 (1.11-1.92)	1.43 (1.04-1.83)	1.56 (1.17-1.92)	1.65 (1.23-2.11)
Monocyte count (×10^9^/L)	0.45 (0.35-0.58)	0.43 (0.34-0.55)	0.43 (0.34-0.54)	0.53 (0.41-0.69)
Platelet count (×10^9^/L)	223 (180-274)	232 (183-290)	213 (178-261)	219 (177-265)
C-reactive protein (mg/l)	2.07 (0.60-8.66)	2.80 (0.97-12.10)	1.89 (0.66-6.15)	0.50 (0.50-6.33)
D-dimer, mg/L	0.41 (0.19-0.86)	0.42 (0.19-0.86)	0.42 (0.23-0.93)	0.24 (0.10-0.68)
Alanine aminotransferase, U/L	22.5 (14.3-37.2)	24.3 (15.3-40.9)	20.8 (13.6-33.8)	21.6 (13.8-35.2)
Aspartate aminotransferase, U/L	20.0 (15.9-26.9)	20.2 (15.9-27.7)	19.1 (15.4-25.2)	21.3 (17.0-27.8)
Albumin, g/L	37.8 (34.7-40.4)	36.8 (33.8-39.5)	38.9 (36.2-41.2)	38.1 (34.5-40.8)
Total bilirubin, μmol/L	9.9 (7.6-12.6)	9.3 (7.2-12.1)	9.8 (7.4-12.8)	10.6 (8.6-13.2)
Blood glucose, mmol/L	5.02 (4.55-5.77)	4.86 (4.44-5.65)	4.90 (4.50-5.63)	5.36 (4.91-6.05)
Urea, mmol/L	4.58 (3.73-5.55)	4.28 (3.50-5.33)	4.48 (3.69-5.71)	4.99 (4.28-5.75)
Creatinine, μmol/L	62.2 (52.7-74.4)	64.0 (54.7-75.2)	64.4 (55.6-76.0)	54.7 (45.3-68.0)
Lactate dehydrogenase, U/L	171.5 (144.7-208.0)	182.7 (155.6-229.0)	175.4 (141.2-221.2)	171.5 (149.7-202.1)

All continuous variables were presented using the median/interquartile range (IQR) values.

### Prognostic factors of in-hospital mortality of COVID-19

Table 2 presents the results of the univariate and multivariate COX proportional hazards regression analysis of in-hospital mortality of COVID-19 in the development cohort of 1780 cases (47 deaths). First, univariate Cox regression analysis was used to explore the potential prognostic predictors (including demographic, symptoms at admission, coexisting disorders, and Laboratory findings at admission), and revealed 29 of the 48 predictors was significantly associated with in-hospital mortality of COVID-19. The HRs ranged from 1.10 (1.07-1.13) for per year increment of age, to 23.11 (9.05-58.98) for lactate dehydrogenase (≥250 U/L). The top five prognostic predictors were lactate dehydrogenase (≥250 U/L, HR:23.11), C-reactive protein (>10mg/l, HR: 12.34), D-dimer (≥0.5 mg/L, HR: 11.29), urea (>7.5 mmol/L, HR: 9.93), and cardiovascular disease (HR: 8.87), respectively.

**Table 2:** Univariate and multivariate COX proportional hazards regression analysis of fetal outcome of COVID-19 in the development cohort.

**Variables**	**Univariate COX analysis**			**Multivariate COX analysis**	
	**HR (95% CIs)**	**P value**		**HR (95% CIs)**	**P value**
Gender, male	2.00 (1.08-3.69)	0.027		-	-
Age, per year increment	1.10 (1.07-1.13)	<0.001		1.05 (1.01-1.09)	0.003
Severity at admission, per rank	8.50 (5.37-13.53)	<0.001		2.91 (1.71-4.97)	<0.001
Respiratory rate (times), ≥30	7.59 (3.29-17.50)	<0.001		-	-
**Symptoms**					
Fever	4.25 (1.03-17.56)	0.045		-	-
Cough	1.54 (0.60-3.96)	0.371		-	-
Fatigue	1.24 (0.60-2.57)	0.561		-	-
Anorexia	1.71 (0.78-3.78)	0.183		-	-
Short breath	1.84 (0.97-3.49)	0.064		-	-
Myalgia	1.17 (0.61-2.25)	0.628		-	-
Chest tight	1.37 (0.71-2.62)	0.346		-	-
Expectoration	0.83 (0.36-1.89)	0.651		-	-
Diarrhea	0.61 (0.15-2.54)	0.495		-	-
Dyspnea	5.59 (3.01-10.39)	<0.001		2.18 (1.11-4.27)	0.023
Sore throat	1.39 (0.33-5.78)	0.652		-	-
Nausea	2.29 (0.70-7.46)	0.171		-	-
Vomiting	1.74 (0.42-7.23)	0.448		-	-
Chest pain	0.87 (0.12-6.38)	0.891		-	-
Dizziness	1.80 (0.44-7.45)	0.417		-	-
**Coexisting disorders**					
Hypertension	3.14 (1.74-5.68)	<0.001		-	-
Diabetes	1.80 (0.95-3.42)	0.074		-	-
Coronary heart disease	4.92 (2.55-9.49)	<0.001		-	-
Cardiovascular disease	8.87 (4.99-15.77)	<0.001		3.25 (1.71-6.17)	<0.001
Cerebrovascular disease	4.44 (1.97-10.00)	<0.001		-	-
Tumor	0.60 (0.08-4.42)	0.618		-	-
Bronchitis	1.00 (0.14-7.33)	0.996		-	-
COPD	5.17 (1.25-21.39)	0.023		-	-
Respiratory disease	2.20 (0.68-7.19)	0.190		-	-
Chronic kidney disease	5.06 (2.14-11.97)	<0.001		-	-
Chronic liver disease	2.38 (1.11-5.14)	0.027		-	-
**Laboratory findings**					
White-cell count (×10^9^/L)					
<4	Referent				
4-10	0.78 (0.30-2.04)	0.611		-	-
≥10	4.42 (1.58-12.33)	0.005		-	-
Neutrophil count (×10^9^/L)					
<1.8	Referent			-	-
2.8-3.6	0.30 (0.06-1.54)	0.149		-	-
≥3.6	1.60 (0.38-6.70)	0.518		-	-
Lymphocyte count, <1.5×10^9^/L	5.84 (2.08-16.43)	0.001		-	-
Monocyte count, ≥0.6 ×10^9^/L	1.28 (0.65-2.54)	0.473		-	-
Platelet count <150 ×10^9^/L	4.46 (2.49-8.02)	<0.001		-	-
C-reactive protein, >10mg/l	12.34 (4.84-31.51)	<0.001		-	-
D-dimer, ≥0.5 mg/L	11.29 (3.48-36.63)	<0.001		-	-
Alanine aminotransferase, >40 U/L	1.46 (0.79-2.69)	0.225		-	-
Aspartate aminotransferase, >40 U/L	3.18 (1.70-5.97)	<0.001		-	-
Albumin, <40 g/L	7.34 (1.00-53.66)	0.049		-	-
Total bilirubin, >17.1 μmol/L	4.50 (2.40-8.43)	<0.001		2.25 (1.18-4.30)	0.014
Blood glucose, >6.1 mmol/L	5.98 (3.25-10.99)	<0.001		2.56 (1.34-4.90)	0.004
Urea, >7.5 mmol/L	9.93 (5.50-17.95)	<0.001		2.14 (1.15-3.97)	0.016
Creatinine, ≥133 μmol/L	4.50 (1.38-14.67)	0.012		-	-
Alkaline phosphatase, >135 U/L	4.15 (1.88-9.17)	<0.001		-	-
γ-glutamyl transpeptidase, >45 U/L	2.46 (1.08-5.60)	0.032		-	-
Creatine kinase, ≥200 U/L	5.92 (2.64-13.29)	<0.001		-	-
Lactate dehydrogenase, ≥250 U/L	23.11 (9.05-58.98)	<0.001		4.53 (1.62-12.63)	0.004

### Construction of the prognostic nomogram

**Figure 1:**
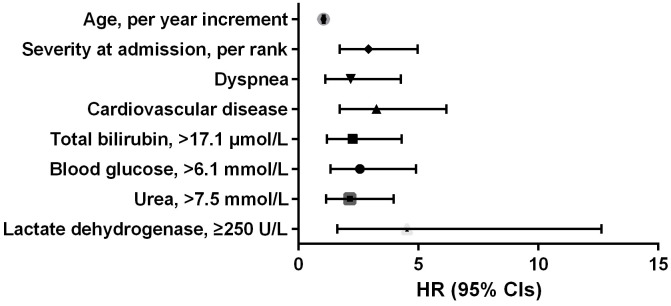
**Independent prognostic predictors associated with in-hospital mortality of COVID-19 in the development cohort.**

**Figure 2:**
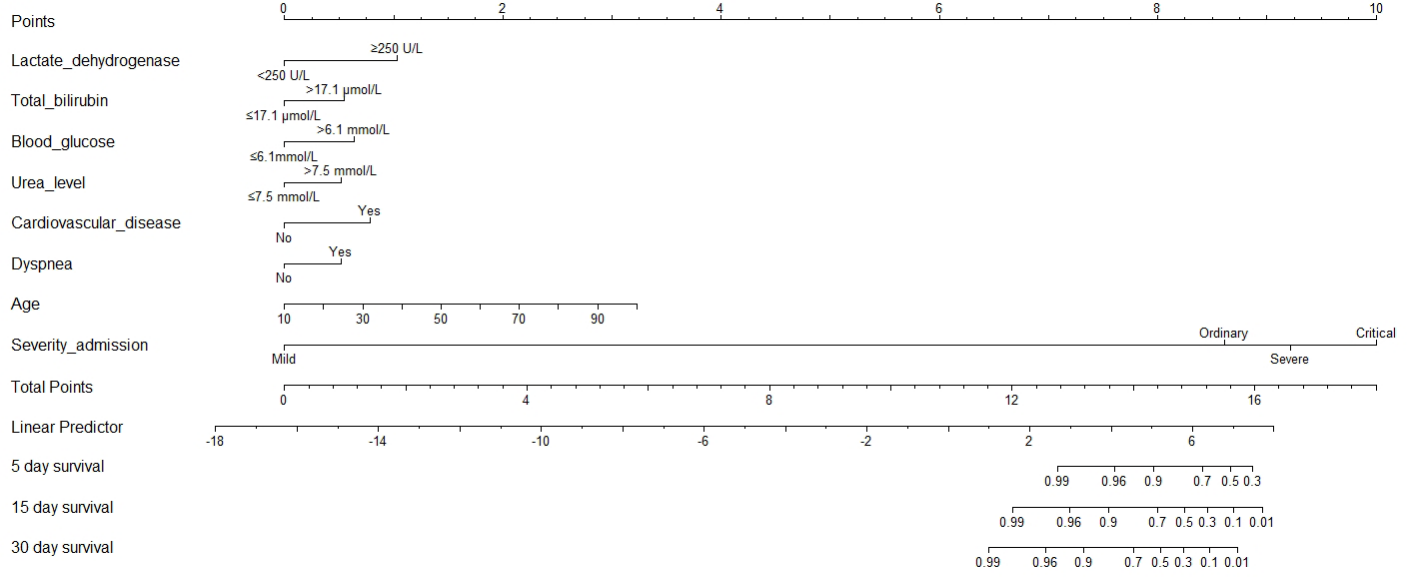
**Prognostic nomogram for COVID-19.** The nomogram variables include age, disease severity at admission, dyspnea, cardiovascular disease, C-reactive protein, total bilirubin, blood glucose, and urea. To use the nomogram, an individual patient’s value is located on each variable axis, and a line is drawn upward to determine the number of points received for each variable value. The sum of these numbers is located on the Total Points axis, and a line is drawn downward to the survival axes to determine the likelihood of survival of 5-, 15-, and 30-day survival.

**Figure 3:**
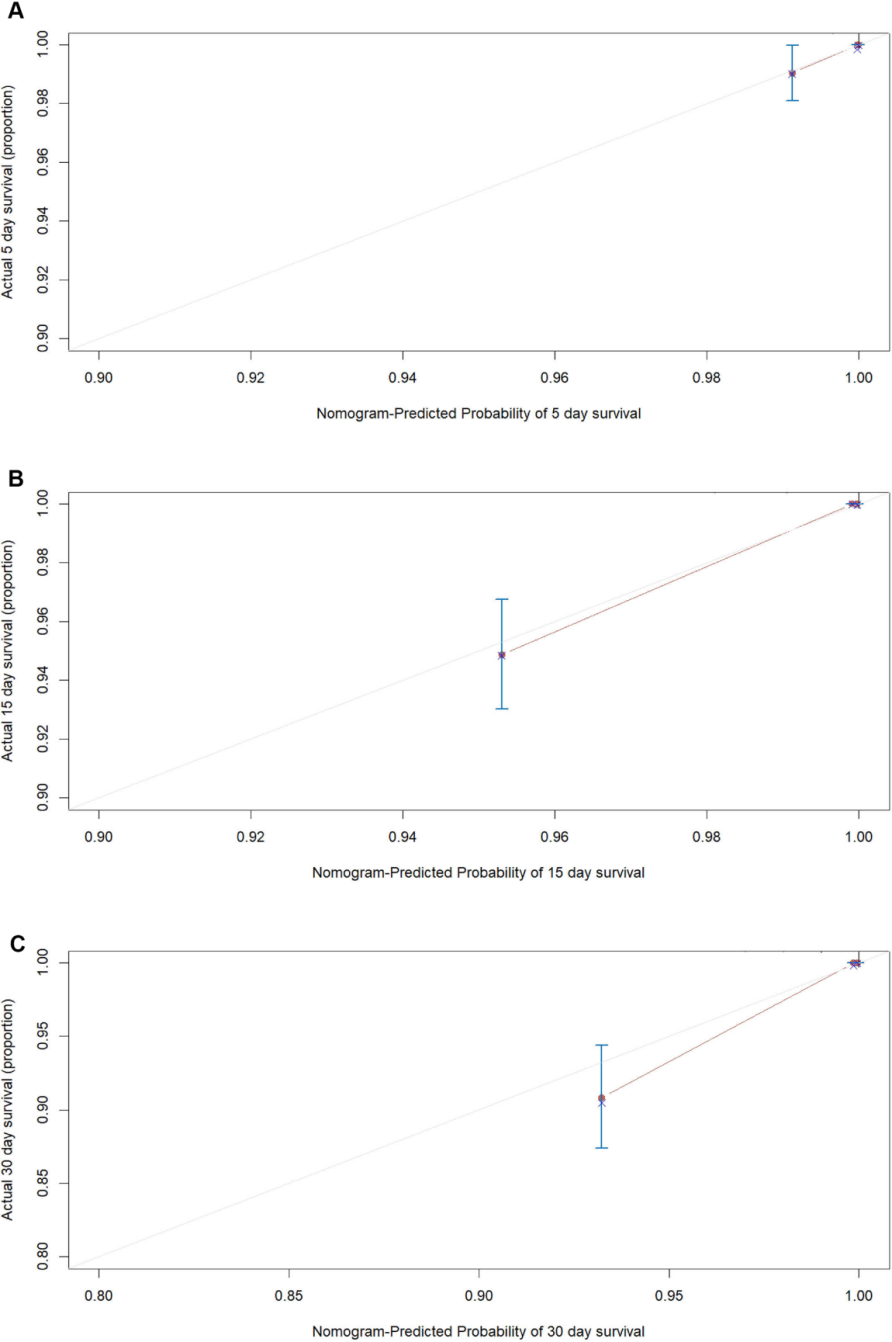
**The calibration plots for the probability of in-hospital mortality of COVID-19 in the development cohort.** Calibration plots of the nomogram predict (**A**) 5-day, (**B**) 15-day and (**C**) 30-day in-hospital mortality in COVID-19 patients in the development cohort. Nomogram-predicted probability of in-hospital mortality is plotted on the x-axis; actual in-hospital mortality is plotted on the y-axis.

According to the AIC selection procedure, 8 independent prognostic predictors (Figure 1), including age (HR for per year increment: 1.05; 95% CIs: 1.01-1.09; P=0.003), severity at admission (HR for per rank increment: 2.91; 95% CIs: 1.71-4.97; P<0.001), dyspnea (HR: 2.18; 95% CIs: 1.11-4.27; P=0.023), cardiovascular disease (HR: 3.25; 95% CIs: 1.71-6.17; P<0.001), lactate dehydrogenase (HR: 4.53; 95% CIs: 1.62-1.63; P=0.004), total bilirubin (HR: 2.56; 95% CIs: 1.34-4.90; P=0.014), blood glucose (HR: 2.56; 95% CIs: 1.34-4.90; P=0.004), and urea (HR: 2.14; 95% CIs: 1.15-3.97; P=0.016) were selected for the construction of the prognostic nomogram (Figure 2). The C-index for in-hospital mortality prediction of COVID-19 was 0.97 (95% CI, 0.95 to 0.98, P<0.001). The calibration plot for the probability of survival at 5-, 15-, and 30-days after admission showed an optimal agreement between the prediction by nomogram and actual observation (Figure 3).

### Validation and calibration of the prognostic nomogram

**Figure 4:**
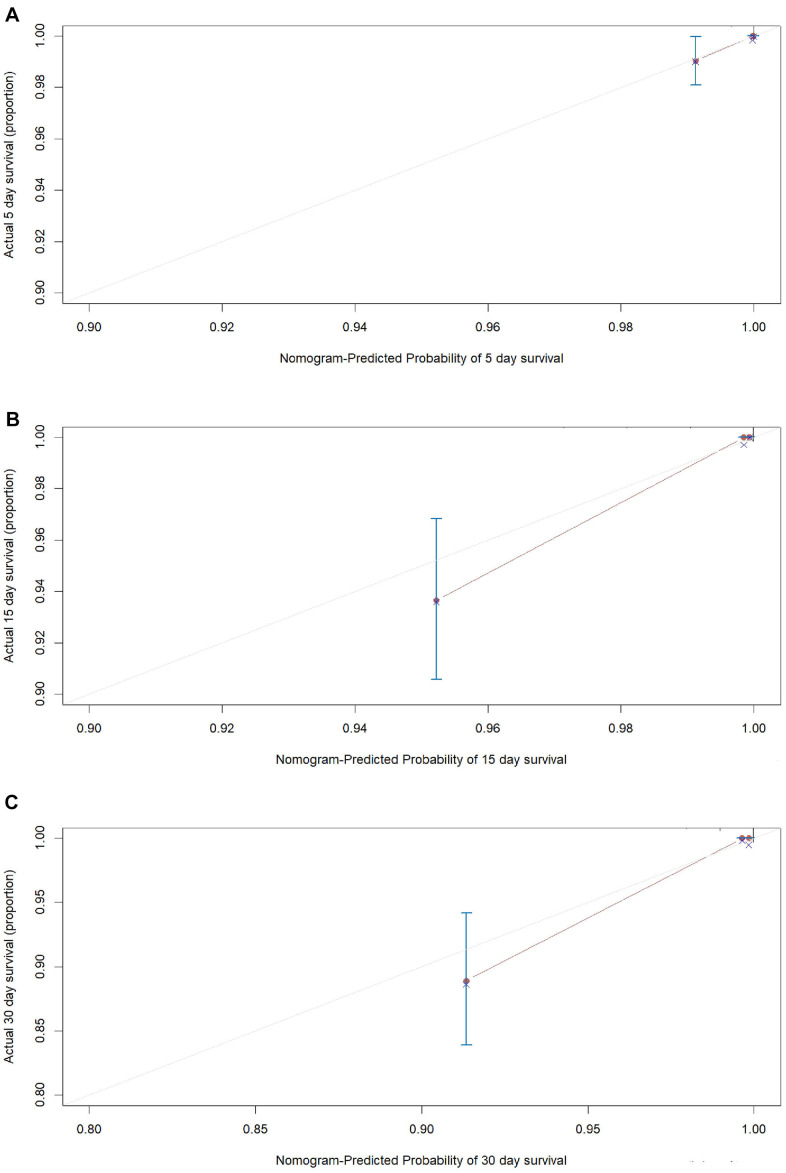
**The calibration plots for the probability of in-hospital mortality of COVID-19 in internal validation cohort.** Calibration plots of the nomogram predict (**A**) 5-day, (**B**) 15-day and (**C**) 30-day in-hospital mortality in COVID-19 patients in the validation cohort. Nomogram-predicted probability of in-hospital mortality is plotted on the x-axis; actual in-hospital mortality is plotted on the y-axis.

**Figure 5:**
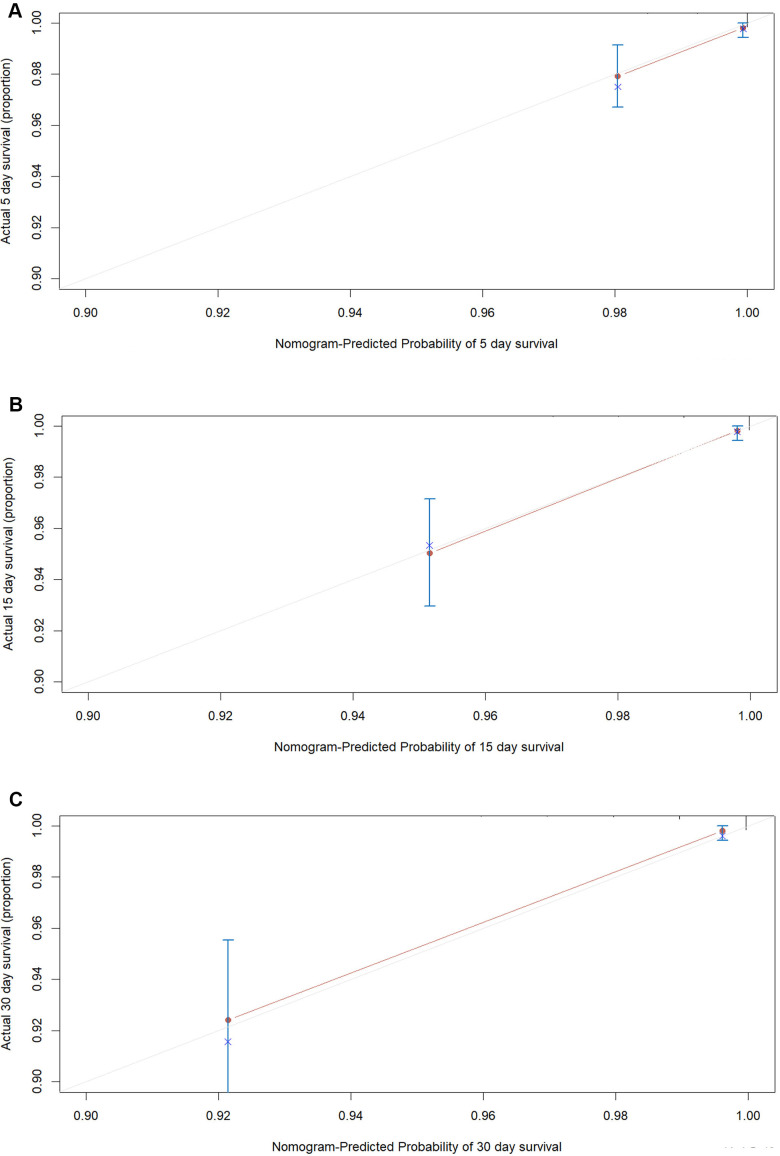
**The calibration plots for the probability of in-hospital mortality of COVID-19 in external validation cohort.** Calibration plots of the nomogram predict (**A**) 5-day, (**B**) 15-day and (**C**) 30-day in-hospital mortality in COVID-19 patients in the validation cohort. Nomogram-predicted probability of in-hospital mortality is plotted on the x-axis; actual in-hospital mortality is plotted on the y-axis.

An internal validation of the prognostic nomogram was conducted in 1,242 COVID-19 cases (22 deaths), while an external validation was conducted in 1,064 COVID-19 cases from Taikang-Tongji hospital (29 deaths). The C-index for in-hospital mortality prediction of COVID-19 was 0.96 (95% CI, 0.94 to 0.98, P<0.001) for the internal validation. For the external validation, the C-index for in-hospital mortality prediction of COVID-19 was 0.92 (95% CI, 0.86 to 0.98, P=9.7×10^-38^). The calibration plot of both internal validation (Figure 4) and external validation (Figure 5) for the probability of survival at 5-,15-, and 30-days after admission also showed an optimal agreement between the prediction by nomogram and actual observation.

## DISCUSSION

In the current study, we established a prognostic nomogram to predict in-hospital mortality of COVID-19 based on eight independent factors including age, severity at admission, cardiovascular disease, and levels of lactate dehydrogenase, total bilirubin, blood glucose, and urea in a large and well-described population of 4, 086 patients. The prognostic nomogram has been validated by internal 1,000 bootstrap resampling, an internal validation, as well as an external validation cohort, maintaining an adequate calibration and discrimination capacity. To our knowledge, this is the largest and most comprehensive study which aims to establish and validate an effective prognostic nomogram for predicting in-hospital mortality of COVID-19 patients to date.

Vague reporting of study population, as well as substantial sampling bias and limited sample size are main obstacles preventing clinical use of previous prognostic models for COVID-19 [12]. The wide variation is mainly caused by excluding participants who did not reach endpoints (neither recovered nor died) and difficulty of data collection under epidemic conditions, with death percentage varying between 1% and 59% in those studies that developed prognostic models to predict mortality [12]. This will inevitably yield a highly selected and biased sample and restrain application of those models. In the current study, we included all patients that had been treated from 2 designated hospitals in Wuhan, with a relatively large cohort of 4, 086 in-hospital patients with 100% ascertainment of endpoints (recovered or died). The clinical characteristics of these patients were well-described, and they serve as a good representation of general in-hospitalized COVID-19 patients.

Demographic, clinical, and laboratory parameters were included in prognostic models for COVID-19. The current model included eight predictors (age, severity at admission, dyspnea, cardiovascular disease, and levels of lactate dehydrogenase, total bilirubin, blood glucose, and urea). Of them, age and comorbidity with cardiovascular disease have been reported in previous models to be risk factors for either mortality or disease progression [9, 13–16]. Severity at admission was mainly determined by SpO2 and CT imaging, as detailed before [17]. The prevalence of dyspnea is barely higher in patients who develop acute respiratory distress and have the poorest clinical outcomes, and was suggested to be a risk factor for predicting mortality in patients with COVID-19 [18, 19].

As for laboratory markers in the current model, the involvement of total bilirubin, blood glucose, urea, and lactate dehydrogenase indicates that involvement of multi-organ dysfunction represents a major predictor of in-hospital mortality for COVID-19 patients. In another recent nomogram, high direct bilirubin level was found to be an independent predictor of 28-day mortality in adult hospitalized patients with confirmed COVID-19 [7]. However, another study with a larger sample size found that AST abnormality, rather than bilirubin, was strongly associated with COVID-19 mortality risk [20]. A recent meta-analysis concluded that comorbid diabetes was associated with an increased risk of disease severity or death in Chinese COVID-19 patients, while it is still not clear to what extent diabetes independently contributes to the increased risk [21]. Besides, acute kidney injury is associated with severe infection and fatality in patients with COVID-19 [22]. The combination of blood urea nitrogen and D-Dimer were predictors of in-hospital mortality in 305 COVID-19 patients, with 27.9% mortality [23].

The current study provides a practical quantitative prognosis judgement tool (nomogram) for clinicians. We have the following strengths: First, sampling bias was avoided as much as possible by inclusion of all COVID-19 patients treated in the 2 designated hospitals, with the largest sample size to date. Second, the model showed good performance in both internal and external validations. Third, C-index and the calibration plot showed adequate calibration and discrimination capacity. Finally, we conducted the current study in strict compliance with the TRIPOD guideline, and PROBAST categorized it as at low risk of bias. Meanwhile, the current study has also several limitations. First, the retrospective study design limited the hierarchy of research evidence, and a prospective study is warranted to confirm the reliability of the findings. Second, missing data of some variables existed due to the emergency situations. However, the missing rate was of less than 10.0%, and the missing values was imputed by EM method. Third, further validations from different hospitals or countries are warranted.

## CONCLUSIONS

Conclusively, a novel prognostic nomogram for COVID-19 based on age, severity at admission, cardiovascular disease, and levels of lactate dehydrogenase, total bilirubin, blood glucose, and urea, was established and validated. It would be helpful for physicians to make optimal treatment decisions, conduct reasonable triage of patients, and avoid delays in treatment. Further studies are warranted to validate whether use of this prognostic nomogram will improve clinical care and patient outcomes of COVID-19.

## MATERIALS AND METHODS

### Patients and study design

The retrospective cohort study for prognosis model of COVID-19 was conducted according to the TRIPOD reporting guideline and the risk of bias was accessed using the PROBAST scales [24–27]. The study protocol was registered in the ChiCTR (http://www.chictr.org.cn, ChiCTR2000030256), and approved by the ethics committee of Wuhan Huoshenshan Hospital (HSSLL024) and Taikang-Tongji hospital (TKTJKY2020146) (Supplementary Materials). The informed consent was waived due to dealing with urgent public health concerns. A total of 3,022 COVID-19 cases from Wuhan Huoshenshan Hospital, and 1,064 COVID-19 cases from Taikang-Tongji hospital with relevant epidemiological and clinical data were included in this investigation. The diagnosis of COVID-19 patients was based on the World Health Organization interim guidance [28]. The endpoint of this study was in-hospital mortality of COVID-19 patients. The degree of severity of COVID-19 at admission was determined according to “Guidance for COVID-19 Prevention, Control, Diagnosis and Management” by National Health Commission of the People’s Republic of China, which was divided into four categories: mild, ordinary, severe and critical (Supplementary Materials) [17]. Of the total 3,022 cases from Wuhan Huoshenshan Hospital, data of 1,780 cases recruited between February 3^rd^ and March 5^th^ was used for the development of the prognostic nomogram, while data for 1,242 cases recruited between March 5^th^ and April 10^th^ was used for the internal validation of the established prognostic nomogram. Further, data of 1,064 COVID-19 cases from Taikang-Tongji hospital was used for the external validation.

### Data collection and entry

Information of demographic characteristics and coexisting disorders was telephone-interviewed using a uniformed questionnaire by two trained physicians. The clinical symptoms, laboratory characteristics, and outcomes information were extracted from the electronic medical records. We double entered and validated the data using EpiData (version 3.1, EpiData Association, Odense, Denmark) software, and disputes were arbitrated by the expert committees composed of experts of respiratory and critical care medicine, and epidemiology.

### Construction of the prognostic nomogram

All statistical analyses were conducted using the SAS statistical software (version 9.4; SAS Institute Inc., Cary, NC, USA) and the R software version 4.0.0 (Institute for Statistics and Mathematics, Vienna, Austria). *P*-value of < 0.05 was considered statistically significant. Categorical variables were described using frequency rates and percentages, while continuous variables were described using the median/interquartile range (IQR) values. The missing values of all potential predictors (missing rate of less than 10.0%) were imputed by expectation-maximization (EM) method. Univariate and multivariate Cox regression analysis was adopted for the estimation of hazard ratio (HR) and corresponding confidence interval (CI) of each variable. First, univariate Cox regression analysis was used to screen the potential prognostic factors which reached a P value of less than 0.05. Then, the independent prognostic factors were derived from a backward stepdown selection process in multivariate Cox regression model. Finally, a prognostic nomogram was formulated based on the results of multivariate analysis by using the rms package, according to the Akaike information criterion (AIC) [29].

### Validation and calibration of the prognostic nomogram

The prognostic nomogram was subjected to 1,000 bootstrap resamples of the primary development cohort, an internal validation cohort, as well as an external validation cohort. The performance of the nomogram was measured by Harrel concordance index (C-index) and the calibration plot. The value of the C-index, which assesses the discrimination of the model, ranges from 0.5 to 1.0, and a larger C-index means a more accurate prognostic model (0.5 indicating a random chance and 1.0 indicating a perfect ability to correctly discriminate the outcome with the model). During the validation of the prognostic nomogram, the total points of each patient in the validation cohort were calculated according to the established nomogram, then Cox regression in this cohort was performed using the total points as a factor, and finally, the C-index and calibration plot were derived based on the regression analysis.

## Supplementary Material

Supplementary Materials
