# Supplementary material for: Development and validation of a prognostic nomogram for predicting in-hospital mortality of COVID-19: a multicenter retrospective cohort study of 4086 cases in China
Source: Aging (Albany NY). 2021 Feb 9;13(3):3176–89. doi: 10.18632/aging.202605 (PMC7906167; doi:10.18632/aging.202605)
Supplement: Supplementary Materials [file aging-13-202605-s001.pdf]

SUPPLEMENTARY MATERIALS

Supplementary Materials

The grading criteria for the severity of COVID-19:

(1) mild type: patients with mild clinical symptoms and no pulmonary changes on CT imaging;

(2) common type: patients with symptoms of fever and signs of respiratory infection, and having pneumonia changes on CT imaging;

(3) severe type: patients presenting with any one of the following conditions: a. respiratory distress, respiratory rate ≥ 30/min; b. oxygen saturation of finger ≤ 93% in resting condition; c. arterial partial pressure of oxygen (PaO2) /oxygen concentration (FiO2) ≤ 300 mmHg (1 mmHg = 0.133 kPa); d. The clinical symptoms are progressively worsening, and lung imaging shows that the lesion has progressed significantly> 50% within 24 to 48 hours;

(4) critical type: patients meeting any one of the following criteria: a. respiratory failure requiring mechanical ventilation; b. shock; c. concomitant failure of other organs and requirement for intensive care unit (ICU) monitoring and treatment.

The relevant ethical review materials

Proposal Title: Epidemiological features, clinical characteristics and prognosis of patients with novel coronavirus pneumonia

Principal investigator: Guoqiang Cao

Study design: observational study

Key requirements:

1. Subjects have the right to be informed about the health effects of the research and the results that can be obtained, and sign informed consent.
2. Each participant's demographic form and consent form will be stored separately in a secure location.
3. Transcription will be carried out in a private space. All personal identification information will be removed or changed during transcription.
4. Digital copies of the files will be encrypted, password protected and stored securely.
5. Research should comply with the Declaration of Helsinki.
